# Introducing SEARCHBreast: a virtual resource to facilitate sharing of surplus animal material developed for breast cancer research

**DOI:** 10.1038/npjbcancer.2016.20

**Published:** 2016-06-29

**Authors:** Bethny Morrissey, Ingunn Holen, Claude Chelala, Phil Carter, Louise Jones, Karen Blyth, Valerie Speirs

**Affiliations:** 1 Leeds Institute of Cancer and Pathology, University of Leeds, Leeds, UK; 2 Academic Unit of Clinical Oncology, University of Sheffield, Sheffield, UK; 3 Barts Cancer Institute, London, UK; 4 Cancer Research UK Beatson Institute, Glasgow, UK

## Abstract

Animals studies have made significant contribution to expanding our knowledge of breast cancer. Often material is leftover and archived. SEARCHBreast provides a platform for collaborative sharing of archived material via a dedicated on-line database whereby users can both share and search available tissue. The SEARCHBreast database has information on over 50 different mouse models, including tissue from PDX models, available to share. With thousands of samples freely available, SEARCHBreast should be the first point of call for any researcher looking for animal material to aid their breast cancer research.

Publication of translational breast cancer research in high-impact journals almost invariably requires extensive *in vivo* experimentation. As a result, many laboratories hold substantial collections of surplus material generated from animal experiments, with only a fraction of this used for the publications relating to the original projects. Despite being developed at a considerable cost, this archival material represents an invisible and largely underutilised resource that often ends up being discarded. Within the breast cancer research community there is both a need and desire to make this valuable material available for researchers to access on a collaborative basis: however, lack of a coordinated system for visualisation and localisation of samples has been a barrier to progress.

SEARCHBreast (**S**haring **E**xperimental **A**nimal **R**esources: **C**oordinating **H**oldings—Breast) was created to overcome this barrier, offering a secure searchable database through which researchers can find, share or upload materials related to animal models of breast cancer, including PDX, genetic and transplantation models.^1,2^ In addition, SEARCHBreast is committed to promoting the use of humanised breast tissue models as replacement alternatives to animals through integration of experts in 3D breast cancer modelling into the SEARCHBreast community^3^ and engagement with a specialist breast cancer biobank, the Breast Cancer Now Tissue Bank.^4^ An integrated bioinformatics portal is under development allowing bioanalysis of mouse/human/cell line ‘omics’ data to help scientists choose the most relevant model to use in their research. Access to SEARCHBreast is freely available to all academic researchers following registration at https://searchbreast.org.
